# The hydrogen sulfide metabolite trimethylsulfonium is found in human urine

**DOI:** 10.1038/srep27038

**Published:** 2016-06-01

**Authors:** Bassam Lajin, Kevin A. Francesconi

**Affiliations:** 1 Institute of Chemistry–Analytical Chemistry, NAWI Graz, University of Graz, Universitaetsplatz 1, 8010 Graz, Austria

## Abstract

Hydrogen sulfide is the third and most recently discovered gaseous signaling molecule following nitric oxide and carbon monoxide, playing important roles both in normal physiological conditions and disease progression. The trimethylsulfonium ion (TMS) can result from successive methylation reactions of hydrogen sulfide. No report exists so far about the presence or quantities of TMS in human urine. We developed a method for determining TMS in urine using liquid chromatography-electrospray ionization-triple quadrupole mass spectrometry (LC-ESI-QQQ), and applied the method to establish the urinary levels of TMS in a group of human volunteers. The measured urinary levels of TMS were in the nanomolar range, which is commensurate with the steady-state tissue concentrations of hydrogen sulfide previously reported in the literature. The developed method can be used in future studies for the quantification of urinary TMS as a potential biomarker for hydrogen sulfide body pools.

Sulfur is the 7^th^ most common element in animals, and represents about 0.2% of human body weight^1^. The functional forms of sulfur are primarily the amino acids methionine and cysteine. Methionine is supplied through the diet as an essential amino acid. Cysteine is also supplied through the diet but it can additionally result from the conversion of methionine via the transsulfuration pathway^2^.

Cysteine can be converted into hydrogen sulfide via three enzymatic pathways^3^,^4^,^5^. Although generally thought of as toxic, H_2_S is attracting growing interest as a signaling molecule^6^, with multiple roles having been recognized in the central nervous system^7^,^8^, cardiovascular system^9^,^10^, and ageing^11^.

Little is known about the regulation of hydrogen sulfide levels and although its mitochondrial oxidation pathways into thiosulfate have been elucidated^12^, some of the enzymes involved have not been fully characterized. One of the pathways that may potentially contribute to the regulation of hydrogen sulfide is the successive methylation reactions via the thiol S-methyltransferase enzyme into dimethylsulfide^13^. Another enzyme, the thioether S-methyltransferase, was shown to convert various thioethers including dimethylsulfide into their respective sulfonium ions in rats^14^,^15^. Mozier *et al.* detected TMS in the urine of rats treated with 250 μmol/kg of dimethylsulfide and [*methyl*-^3^H] methionine^15^, but the endogenous levels of TMS in animals and whether it is significantly produced without administration of the parent thioether is not known. Furthermore, no report exists so far about the presence of TMS in humans.

The aim of the present study was to develop an analytical method for the quantitative measurement of TMS in urine, and to apply the method to determine if TMS is a natural constituent of human urine. We discuss the results in terms of the possible use of urinary TMS as an indicator of hydrogen sulfide levels in humans.

## Results and Discussion

**Figure 1:**
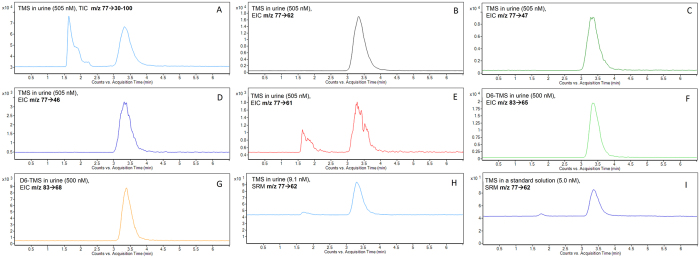
HPLC-MS/MS chromatograms for the detection of TMS and d_6_-TMS. The panes show a chromatogram for the total ion current (TIC) for a product ion scan of the precursor ion m/z 77 (**A**), extracted ion chromatograms (EIC) for its detected fragments ((**B–E**) see Fig. 2), extracted ion chromatograms for the two major fragments of the deuterated internal standard in spiked urine m/z 83→65 and m/z 83→62 (**F,G**), and selected reaction monitoring (SRM) for the detection of low levels of TMS in urine (**H**) and a standard solution (**I**). For quantitation, the mass transitions m/z 77→62 and 83→65 were used for TMS and d_6_-TMS, respectively.

**Figure 2:**
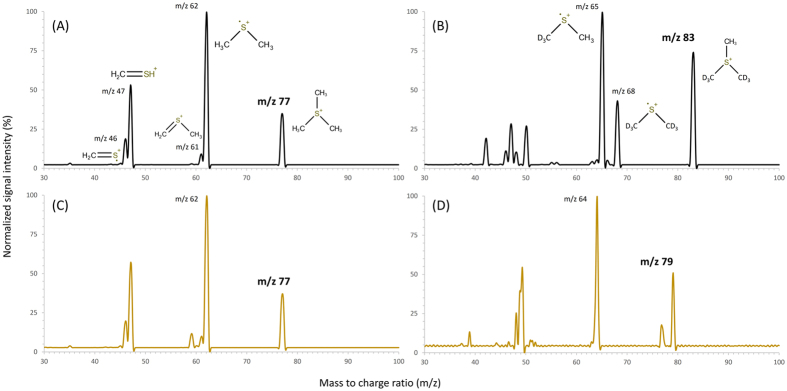
Product ion scan at retention time 3.4 min of (**A**) TMS in water (500 nM); (**B**) the internal standard d_6_-TMS (500 nM) in water; (**C**) endogenous TMS corresponding to the ^32^S isotope in a urine sample containing 505 nM of TMS; (**D**) endogenous TMS corresponding to the ^34^S isotope (relative abundance 4.2%) in a urine sample containing 505 nM of TMS. Peaks labeled with a m/z value in bold represent the precursor ions.

Although an HPLC method for purity analysis of TMS used in organic synthesis has been reported^16^, the method utilizes a conductivity detector, which lacks the high sensitivity and selectivity necessary for determining a trace analyte in complex biological fluids Thus, we developed a HPLC/tandem mass spectrometry method utilizing an isotopically labeled internal standard (d_6_-TMS) to compensate for matrix suppression effects, that allowed the direct quantitative determination of TMS in untreated urine. The mass transition *m/z* 77→62 was selected over other transitions for quantification as it provided low background (Fig. 1) and the highest sensitivity (Fig. 2). The analytical performance indicators of the method, tested using urine samples spanning a wide range of specific gravities, are recorded in Table 1. In brief, the LOD and LOQ values in water were 0.2 and 0.6 nM TMS, respectively, and these values in urine were 2–4 fold higher depending on the various urine matrices; the calibration was linear (r^2^ = 0.9999) up to at least 5000 nM TMS; and the average %RSD of the intra-day and inter-day precision was 5.3 and 6.4%, respectively. Nine urine samples were analyzed using both the method of standard addition and the method of an internal standard (d_6_-TMS), and the results agreed to within 2–16% depending on the urine matrix and TMS concentration (Table 1). During the course of our study, we found TMS to be a stable analyte; urine stored at 4 °C for 6 days returned TMS values 94–103% relative to samples stored at −80 °C.

**Figure 3:**
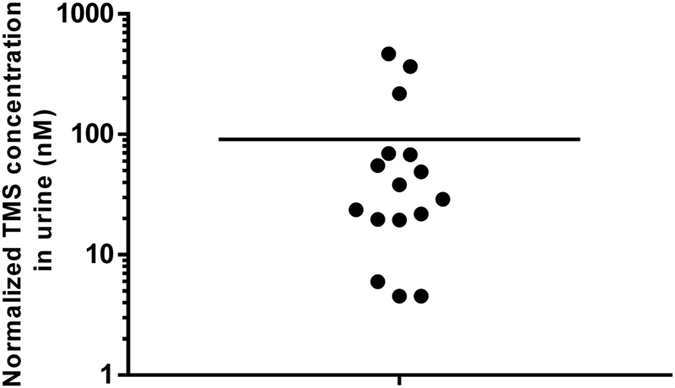
Trimethylsulfonium urinary levels in a group of 16 healthy volunteers. The line represents the average concentration. The concentrations were normalized according to specific gravity based on the equation: C_normalized_ = C_measured_ (SG_average_ − 1)/(SG_sample_ − 1)^17^.

**Figure 4:**
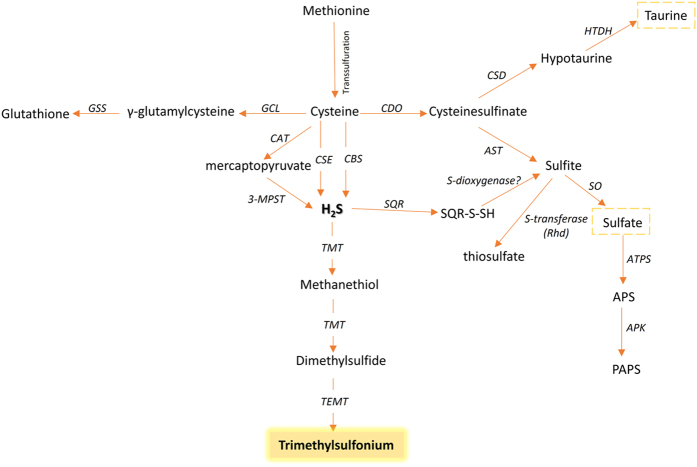
A simplified schematic of the metabolic network of sulfur centered on hydrogen sulfide. Excess cysteine is converted to sulfate (~80% of total urinary sulfur^21^), or taurine (~3% of total urinary sulfur^21^). In addition to the mercaptopyruvate pathway, two enzymes belonging to the transsulfuration pathway (CSE and CBS) undertake the production of hydrogen sulfide. Hydrogen sulfide is oxidized in the mitochondria in a three-step process^12^. First, SQR-bound persulfide is formed at a cysteine residue of SQR. Second, a putative sulfur dioxygenase oxidizes the persulfide group producing sulfite. Third, a sulfur transferase enzyme (rhodanese) transfers a sulfur atom to sulfite from another molecule of SQR-bound persulfide to form thiosulfate. Another source of thiosulfate is the transfer of a sulfur atom from mercaptopyruvate to sulfite via 3-MPST^22^. Sulfate can be activated to form the global sulfate donor PAPS which results in the formation of ester sulfate metabolites that collectively contribute up to ~9% of total urinary sulfur^21^. 3-MPST, 3-mercaptopyruvate sulfurtransferase; APS, adenosine 5′-phosphosulfate; ATPS, ATP sulfurylase; APK, APS kinase; CAT, cysteine aminotransferase; CDO, cysteine dioxygenase; AST, aspartate (cysteinesulfinate) aminotransferase; CSD, Cysteine sulfinic acid decarboxylase; CSE, cystathionine γ-lyase; CBS, cystathionine β-synthase; GCL, glutamate-cysteine ligase; GSS, glutathione synthetase; HTDH, hypotaurine dehydrogenase; PAPS, 3′-phosphoadenosine-5′-phosphosulfate; Rhd, rhodanese; SO, sulfite oxidase; SQR, sulfide quinone reductase; SQR-S-SH, SQR-bound persulfide; TMT, thiol *S*-methyltransferase; TEMT, thioether *S*-methyltransferase.

The concentrations of TMS in the population studied were within the range 2.7–505 nM with a mean of 99 nM and a median of 32 nM. After normalization according to specific gravity^17^, the concentrations were within the range 4.5–467 nM with a mean of 91 nM and a median of 34 nM (Fig. 3). These concentrations are 5–7 orders of magnitude lower than the urinary concentration of total sulfur previously reported^18^, and indicate a negligible role for TMS as an elimination product for excess sulfur or hydrogen sulfide. An interpretation of the possible significance of these low levels of TMS, however, requires a review of the metabolic network of sulfur (Fig. 4). Excess cysteine originating from the diet or from the transsulfuration pathway is first oxidized into cysteine sulfinate, which is finally converted either into sulfate, the primary urinary excretion species of excess sulfur^18^, or taurine. Tissue hydrogen sulfide is produced primarily from cysteine via three enzymatic activities^3^,^4^,^5^ at high rates^19^ that necessitate rapid oxidation into thiosulfate or sulfate, maintaining a steady-state concentration of intracellular hydrogen sulfide at low nanomolar levels (~15 nM in mouse brain and liver)^19^,^20^. These levels of hydrogen sulfide in tissues are in the same range as the nanomolar concentrations of urinary TMS found in the present study (Fig. 3). This observation suggests that urinary TMS levels might serve as a specific indicator for the steady-state levels of tissue hydrogen sulfide, and thus could be associated with medical conditions where hydrogen sulfide signaling is involved^7^,^8^,^9^,^10^.

While thiosulfate, produced at levels around 0.1% of total urinary sulfur^21^, could also be considered as an indicator of hydrogen sulfide levels, it can originate from other substrates (e.g. the sulfur transfer from mercaptopyruvate to sulfite^22^), rendering it non-specific to hydrogen sulfide levels. Furthermore, hydrogen sulfide can be of exogenous origin due to sulfate reduction by the microflora at high rates in the colon^23^. The expression of the enzyme thioether S-methyltransferase which produces TMS is among the lowest in the colon^24^. On the other hand, the highest activity of the hydrogen sulfide oxidation pathways leading to thiosulfate is found in the colonic mucosa^23^, rendering urinary thiosulfate much more heavily dependent on the intestinal hydrogen sulfide exposure than is TMS.

Any potential utility or interpretation of the levels of urinary TMS must consider possible confounding sources of variability. First, one of the formulations of glyphosate, a herbicide commonly used for agricultural purposes, is based on the trimethylsulfonium salt^25^. The existence of this species in food and the significance of its possible contribution to the excretion of TMS in human urine are yet to be investigated. Second, it has recently been discovered that polymorphisms in the gene for indolethylamine N-methyltransferase (another name for thioether S-methyltransferase) are associated with a marked variation (20–40 fold) in the production level of trimethylselenonium ion^26^. Our future work will include an investigation of whether these polymorphisms also affect the sulfur analogue in a similar manner.

## Conclusion

Trimethylsulfonium, a metabolite of hydrogen sulfide, is detected in human urine for the first time. The possible clinical or physiological significance of this metabolite warrants investigation.

## Methods

### Study cohort

The study involved single urine samples from 16 healthy volunteers (7 females, 9 males): the mean age (SD) and age range were 40 (12) and 19–61 years; the mean BMI (SD) and BMI range were 24.3 (2.1) and 21.4–29.3 kg m^−2^. Morning urine (first pass of the day) was collected in a 300 ml sample collection bottle (Corning, NY, USA); the collected urine samples were divided into several portions of ca. 5 ml and stored at −80 °C until analysis. Volunteers gave informed consent to participate in the study, and all procedures were in accordance with the Declaration of Helsinki. All experimental protocols were approved by the University of Graz (GZ. 39/10/63).

### Chemicals and reagents

Trimethylsulfonium iodide was purchased from Sigma-Aldrich (purity > 98.0%, Vienna, Austria). HPLC grade acetonitrile was purchased from BDH chemicals (purity > 99.9%, HiPerSolv CHROMANORM^®^, BDH chemicals, Poole, UK). Ammonium formate (purity > 99.0%) was purchased from Sigma-Aldrich (Vienna, Austria). Ammonia was purchased from Sigma-Aldrich (concentration: ≥ 25% in water, Vienna, Austria). Water (18.2 MΩ) was purified by a Milli-Q water purification system (Millipore Ltd., USA).

Isotopically labeled d_6_-trimethylsulfonium (d_6_-TMS) to be used as an internal standard was synthesized in-house following a previously described procedure^27^. Thus, equimolar amounts of d_6_-dimethylsulfide (324 μL, purity 99%, isotopic purity 99 atom D %, Sigma-Aldrich, Vienna, Austria) and iodomethane (250 μL, purity 99.5%, Sigma-Aldrich, Vienna, Austria) were combined in a capped 10 ml glass test tube; the mixture was left overnight at room temperature whereupon it yielded a white precipitate of d_6_-trimethylsulfonium iodide. An aqueous solution of 100 nM of d_6_-trimethylsulfonium was prepared and used as an internal standard by co-injection with 1 μL using the HPLC autosampler.

### The determination of trimethylsulfonium (TMS) in urine

We developed an HPLC method that utilizes an electrospray ionization-triple quadrupole (ESI-QQQ) mass spectrometric detector. We used a Hamilton PRP-X200 cation-exchange column (2.1 × 150 mm; Hamilton, Reno, NV, USA), with 10 mM ammonium formate including 5% acetonitrile, adjusted with ammonia to pH 9.0 as the mobile phase. The mobile phase flow rate was set to 0.25 mL min^−1^ and the column temperature was set to 30 °C. The injection volume was 1 μL. The triple quadrupole mass analyzer (Agilent jet stream (AJS) ESI-QQQ 6460 system, Agilent Technologies, Waldbronn, Germany) was operated in the multiple reaction monitoring mode (MRM), with a collision energy of 18 eV, a fragmentor voltage of 40 V, and cell accelerator voltage of 7 V. The first quadrupole was set at m/z of 77 and 83 and the third quadrupole at m/z of 62 and 65, for TMS and d_6_-TMS, respectively. The capillary voltage was set to +2 kV. The nebulizer gas pressure was 30 psi. The nebulizer gas flow and temperature were 13.0 L min^−1^ and 300 °C, respectively. The sheath gas flow and temperature were 12.0 L min^−1^ and 400 °C, respectively.

The thawed urine samples were mixed vigorously by shaking and vortexing prior to filtration through 0.2 μm nylon filters, then injected (1 μL) onto the HPLC column without prior dilution or purification. Quantification was based on peak areas within a calibration range of 1–500 nM. The LOD and LOQ were calculated based on the method of the standard error of the y-intercept (3* SEy and 10* SEy for LOD and LOQ, respectively) for a calibration curve recorded at low levels within the range 1–10 nM. Matrix suppression was assessed by spiking water and a series of urine samples spanning a wide range of specific gravity (1.005–1.031) with different levels (25, 100, and 500 nM) of the internal standard. The adjustment of concentrations according to specific gravity was based on the equation C_normalized_ = C_measured_ (SG_average_ − 1)/(SG_sample_ − 1)^17^.

## Additional Information

**How to cite this article**: Lajin, B. and Francesconi, K. A. The hydrogen sulfide metabolite trimethylsulfonium is found in human urine. *Sci. Rep.* **6**, 27038; doi: 10.1038/srep27038 (2016).

## Figures and Tables

**Table 1:**
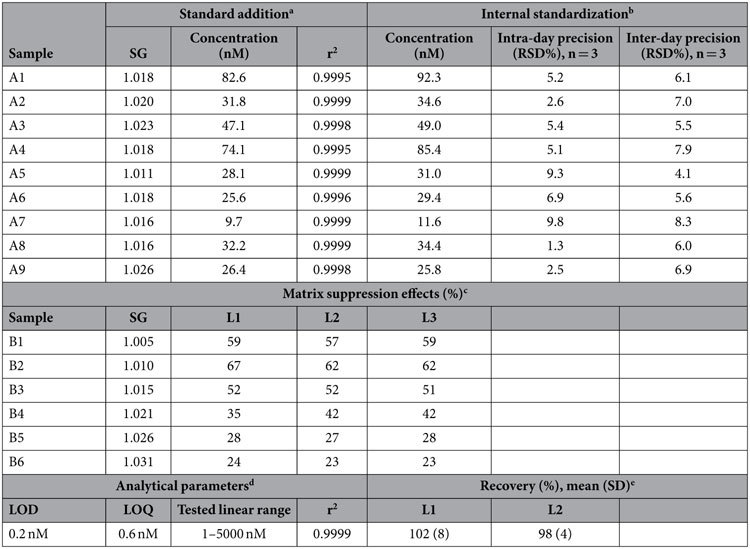
Analytical performance indicators of the HPLC/MS-MS method for TMS.

^a^The standard addition method was applied by co-injecting equal volumes (1 μL) of the urine sample and standard solutions with concentrations of 0, 5, 10, 25, 50, 100, and 250 nM. SG, specific gravity.

^b^The internal standardization method was applied by co-injecting equal volumes (1 μL) of the standards/urine samples and a solution of 100 nM internal standard d_6_-TMS. Note that the inter-day replicates involved repeated freeze-thawing (1 freeze-thaw cycle per replicate).

^c^The matrix effects were studied by co-injecting equal volumes (1 μL) of the urine samples and a solution of the internal standard (d_6_-TMS) at different concentration levels (L1, 25; L2, 100; L3, 500 nM) and comparing the peak area with water co-injection (which is assigned 100%).

^d^The LOD and LOQ values were based on the method of the standard error of the y-intercept (3* SEy and 10* SEy for LOD and LOQ, respectively, for a calibration curve recorded at low levels within the range 1–10 nM). Actual LOD and LOQ values in urine are higher by 2–4 fold and vary depending on the matrix of the urine sample (see matrix suppression effects). The injection volume is 1 μL.

^e^The recovery was calculated by manually spiking a series of different urine samples with either low levels (L1, 50 nM) or high levels (L2, 250 nM) of TMS.
